# Efficacy of a nootropic spearmint extract on reactive agility: a randomized, double-blind, placebo-controlled, parallel trial

**DOI:** 10.1186/s12970-018-0264-5

**Published:** 2018-12-12

**Authors:** Paul H. Falcone, Aaron C. Tribby, Roxanne M. Vogel, Jordan M. Joy, Jordan R. Moon, Chantelle A. Slayton, Micah M. Henigman, Joanne A. Lasrado, Brandon J. Lewis, Brenda A. Fonseca, Kristin M. Nieman, Kelli A. Herrlinger

**Affiliations:** 1 MusclePharm Sports Science Institute, Denver, CO USA; 2 Mercy Hospital, Springfield, MO USA; 3 Gu Energy Labs, Berkeley, CA USA; 4 Department of Nutrition and Food Sciences, Texas Woman’s University, Dallas, TX USA; 5 Impedimed, Inc., Carlsbad, CA USA; 6 Metropolitan State University, Denver, CO USA; 7 Kemin Foods L.C, Des Moines, IA USA; 8 Katalyses, Ankeny, IA USA

**Keywords:** Spearmint, Polyphenols, Rosmarinic acid, Cognition, Reaction time, Agility, Makoto, Nootropic

## Abstract

**Background:**

Proprietary spearmint extract (PSE) containing a minimum 14.5% rosmarinic acid and 24% total phenolic content, has evinced positive effects on cognition in individuals aged 50–70 with memory impairment after chronic supplementation. To address the growing interest in connecting mental and physical performance, the present study examined whether the nootropic effects of PSE translate into changes in reactive agility following daily supplementation with PSE.

**Methods:**

Utilizing a randomized, double-blind, placebo-controlled, parallel design, healthy, recreationally-active men and women (*n* = 142) received 900 mg of PSE or placebo (PLA) daily for 90 days. Reactive agility, our primary outcome, was determined by measuring the number of hits and average reaction time (ART) on a Makoto Arena II, a 360^0^ audio-visual device that measures stationary, lateral, and multi-directional active choice reaction performance. Safety was evaluated using complete blood count, comprehensive metabolic panel, and blood lipids. Measurements were evaluated on days 7, 30, and 90 of supplementation.

**Results:**

An overall treatment effect (*p* = 0.019) was evident for increased hits with PSE on the stationary test with footplates, with between group differences at Day 30 (PSE vs. PLA: 28.96 ± 2.08 vs. 28.09 ± 1.92 hits; *p* = 0.040) and Day 90 (PSE vs. PLA: 28.42 ± 2.54 vs. 27.02 ± 3.55 hits; *p* = 0.002). On the same task, ART improved (treatment effect, *p* = 0.036) with PSE at Day 7 (PSE vs. PLA: 0.5896 ± 0.060 vs. 0.6141 ± 0.073 s; *p* = 0.049) and Day 30 (PSE vs. PLA: 0.5811 ± 0.068 vs. 0.6033 ± 0.055 s; p = 0.049). PSE also significantly increased hits (treatment effect, *p* = 0.020) at Day 30 (PSE vs. PLA: 19.25 ± 1.84 vs. 18.45 ± 1.48 hits; *p* = 0.007) and Day 90 (PSE vs. PLA: 19.39 ± 1.90 vs. 18.66 ± 1.64 hits; *p* = 0.026) for the multi-directional test with footplates. Significant differences were not observed in the remaining Makoto tests. PSE was well tolerated as evidenced by no effects observed in the blood safety panels.

**Conclusions:**

The findings of the current study demonstrate that consumption of 900 mg of PSE improved specific measures of reactive agility in a young, active population.

**Trial registration:**

clinicaltrials.gov, NCT02518165. Registered August 7, 2015 – retrospectively registered.

**Electronic supplementary material:**

The online version of this article (10.1186/s12970-018-0264-5) contains supplementary material, which is available to authorized users.

## Background

Recent evidence indicates that many individuals are increasingly willing to utilize supplements that enhance cognition, known as nootropics, to improve or maintain brain health [1]. While the primary use of nootropics may be for everyday improvements in attention and focus, athletes and active individuals are supplementing with nootropics in an effort to enhance their performance [2–5]. Preliminary evidence indicates that increased focus can improve physical performance [6–9] but there is very little evidence to date indicating that enhanced cognition through supplementation results in improved athletic performance.

Many botanicals have demonstrated nootropic benefits following oral administration, such as gingko biloba [10], ginseng [11], and various members of the mint (Lamiaceae) family [12–15]. Traditionally, members of the mint family have been utilized for nervous system disorders, as well as respiratory and gastrointestinal issues [16–18], due to active ingredients such as menthone, piperitone oxide, camphor, linalool, and rosmarinic acid [19, 20]. Rosmarinic acid is a caffeic acid ester found in rosemary and spearmint that has demonstrated antimicrobial [21], antiviral [22], antioxidant [21, 23], anti-inflammatory [24], and neuroprotective [25–31] activity in vitro and in vivo. More recently, botanicals in the mint family, such as sage [12–14], rosemary [15], and lemon balm [32], have been shown to benefit memory (speed of memory and secondary) and attention. The proprietary spearmint extract (PSE) utilized in the present study was developed through traditional breeding techniques to contain more than 60 phenolic compounds in addition to higher amounts of naturally-occurring rosmarinic acid (a minimum of 14.5%) than other commercially available spearmint extracts [33]. An open-label human trial showed that 900 mg of PSE for 30 days significantly improved reasoning and attention in individuals aged 50–70 with self-reported, age-related memory issues [34]. A randomized, double-blind, placebo-controlled study was conducted in individuals with age-associated memory impairment using PSE supplemented for 90 days [35] and demonstrated significant improvement in both quality of working memory and spatial working memory. Finally, small-scale studies in younger populations, aged 18–50, were conducted which showed that acute consumption of PSE numerically improved subjective assessment of cognitive improvements and objective measures of attention [36]. Though studies have investigated Lamiaceae and cognition, less research has been conducted on spearmint consumption and exercise performance.

Essential oils of Lamiaceae, specifically spearmint and peppermint, have been shown to have positive effects on lung function; therefore, studies have investigated their effects on exercise performance. An open-label study demonstrated that inhalation of *Mentha spicata* improved lung function and running times of healthy individuals [37]. Similarly, Meamarbashi and colleagues [38] observed that healthy university students improved their ventilatory outcomes, running time to exhaustion, work, and power after 10 days of consumption of 50 μl of peppermint. In a follow-up study by the same laboratory utilizing the same population [39], a single dose of 50 μl of peppermint increased grip force, vertical jump, and long jump. Additionally, the researchers tested simple reaction time via a passive computerized test battery, and both audio simple reaction time and visual reaction time improved [39]. Though the effects of Lamiaceae on exercise performance and reaction time have been tested within a single study, one cannot draw conclusions linking physical and mental performance since these observed benefits may have been caused by separate mechanisms, such as improved lung function and increased focus, respectively. Preliminary data from our laboratory indicated that an intervention can differentially affect results from active and passive choice reaction performance testing [40], suggesting that passive choice reaction testing may not necessarily translate to performance in athletic endeavors. Active choice reaction performance can translate to athletic performance by enhancing an athlete’s reactive agility, which can be defined as any unplanned change in direction or speed. Reactive agility is an aspect of physical performance; interestingly, however, data suggest that reactive agility may rely more on cognitive than physical parameters [41]. In fact, reactive agility correlated more highly with response time and decision-making time – which are aspects of cognition – than sprint speed, change-of-direction speed, or other physical measurements [41]. In order to link mental and physical performance, novel testing devices that combine physical and cognitive activities, like the Makoto device, may be useful. Therefore, the purpose of the present study was to investigate the effects of daily supplementation of 900 mg of PSE, a water extracted proprietary spearmint extract with a robust phenolic profile, on reactive agility. The primary outcome of the study was reactive agility, as measured by the Makoto Arena II device. Secondary outcomes included inflammation, stress, and safety as measured by analyses of blood biomarkers. The research hypothesis was that daily supplementation with 900 mg of PSE will significantly improve reactive agility compared to placebo.

## Methods

### Experimental design

**Fig. 1:**
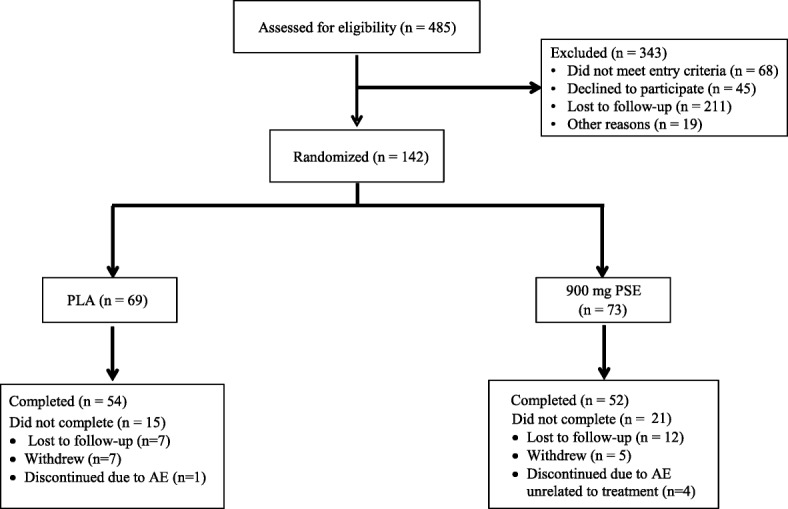
CONSORT diagram for Study Participants. A total of 485 participants were screened. Healthy active young men and women were randomly assigned to one of two treatments, 0 PLA or 900 mg/day PSE (Total randomized *N* = 142; *N* = 69 (PLA), *N* = 73 (PSE)). A total of 54 and 52 subjects completed the trial in the PLA and 900 mg/day PSE groups, respectively. Abbreviations: PSE, proprietary spearmint extract; PLA, placebo; AE, adverse event

**Fig. 2:**
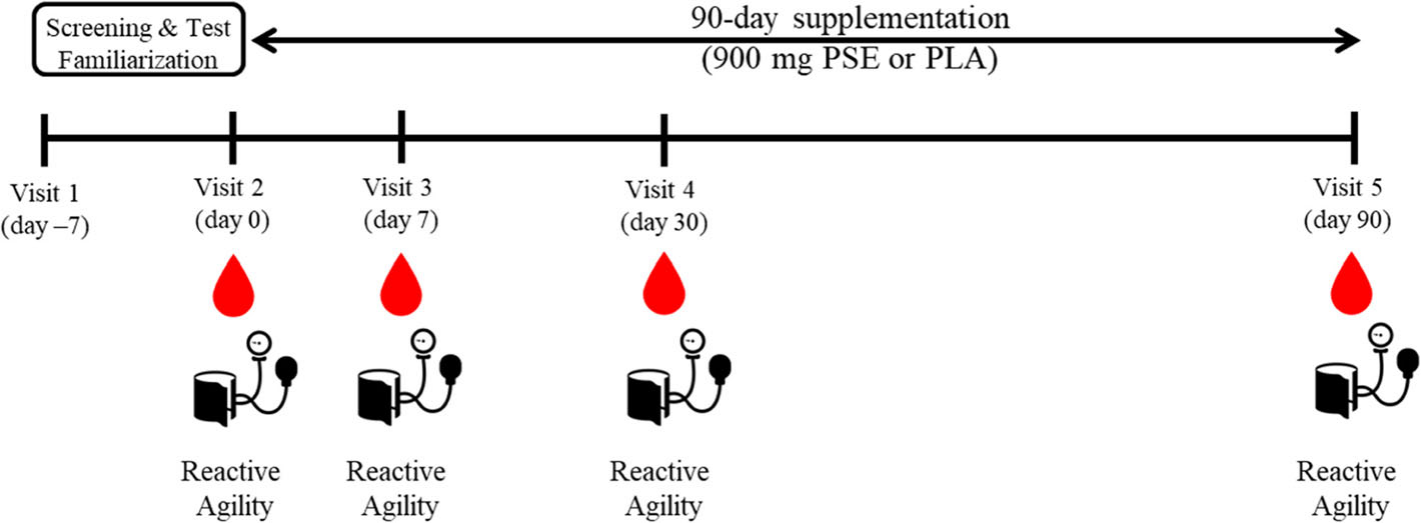
Experimental study design. Abbreviations: PSE, proprietary spearmint extract; PLA, placebo

A randomized, double-blind, placebo-controlled, parallel design was implemented to determine the effects of PSE on reactive agility in healthy individuals aged 18–50 years. A total of 485 individuals were initially contacted via phone to screen for eligibility (Fig. 1). Individuals who met inclusion/exclusion criteria were asked to report to the laboratory (MusclePharm Sports Science Institute, Denver, CO) for a familiarization visit so that the reactive agility tests could be practiced to minimize any learning effects (Fig. 2). One hundred forty-two participants were randomized into PSE or placebo (PLA) groups in a staggered start design (1 week after screening visit). To generate a randomization schedule using block randomization, participants were stratified by sex and age into four groups: young (18–35 years)/male, older (36–50 years)/male, young/female, and older/female. A random sequence of blinded treatments (A and B) was generated by the principal investigator using 37 blocks of 4 on a research randomizer website (www.randomizer.org). These blocks were applied to each stratum mentioned above as each previous block of 4 was filled by participants. All blood was collected at a LabCorp facility and all other measures were performed at the MusclePharm Sports Science Institute.

At baseline (Day 0) participants were instructed to take two capsules of either PLA or PSE (providing 900 mg PSE/day containing a minimum of 14.5% rosmarinic acid and 24% total polyphenols (expressed as gallic acid equivalents); Neumentix™ Phenolic Complex K110–42, Kemin Foods, L.C., Des Moines, IA) each morning with breakfast for 90 days, except on test days, when they took the capsules directly after testing. Both types of capsules were produced by the same manufacturer to be identical in shape, size, and color, and were sealed in identical bottles. All investigators involved in product dispensing, data collection, and analysis of outcomes were blinded in the following manner: PSE and PLA bottles were labeled “A” or “B” by unblinded individuals at Kemin Foods LC. who were not involved in subject interaction or data assessment. All bottles were labeled according to ICH-GCP guidelines. At each post-supplementation study visit (Days 7, 30, and 90) capsules were counted for compliance. Daily compliance was also logged by the participants in a study diary as a secondary measure. After fasting for 10 h, participants arrived at the laboratory at baseline, and after 7, 30, and 90 days of consuming the supplement daily. Testing consisted of an interactive audio-visual reaction time test battery, a blood draw, and vital signs assessment. After testing, a meal replacement bar was provided to participants (Combat Crunch™; MusclePharm Inc., Denver, CO). An Institutional Review Board (Quorum Review IRB, Seattle, WA) approved the study protocol and informed consent documents prior to initiation of the study. Furthermore, all study procedures were consistent with Good Clinical Practices under the United States Title 21 of the Code of Federal Regulations and the Declaration of Helsinki.

### Participants

Healthy, recreationally-active men and women between the ages of 18–50 years were recruited for the present study. Healthy was defined as having a body mass index between 18.5–29.99 kg/m^2^ or between 30.0–34.99 kg/m^2^ with a body fat of < 39% for women aged 18–39 years, < 40% for women aged 40–50 years, < 25% for men aged 18–39 years, or < 28% for men aged 40–50 years [42]. Body fat was determined using bioelectric impedance analysis (InBody Co., Seoul, South Korea). Health status was assessed using a health history questionnaire. A recreationally-active individual was defined as ≥1 and ≤ 6 h of moderate and/or vigorous exercise per week as measured by the New Zealand Physical Activity Questionnaire [43]. Eligibility also included willingness to abstain from caffeine-containing products for 10 h prior to and during all test visits, from alcohol consumption and physical activity for the previous 24 h, and from strenuous resistance exercise (defined as heavier than a 6-repetition maximum [the most weight which a participant could successfully lift 6 times]) for 48 h. Participants were required to keep their sleep consistent (within 2 h) each night prior to all study visits. Finally, participants were required to maintain their normal diet (including repetition of dietary intake for the 24 h prior to each study visit), exercise, and sleep regimens throughout the study. All females were amenorrhoeic during test visits. A high school diploma or the equivalent was also necessary for inclusion.

Participants were excluded from the study if they had a history or presence of a clinically important cardiac, renal, hepatic, endocrine (including diabetes mellitus), pulmonary, biliary, gastrointestinal, pancreatic, uncontrolled hypertension, depression or neurologic disorder. Presence of cancer in the past 2 years was a criterion for exclusion. Additional criteria for exclusion included: colorblindness, pregnancy, history of alcohol abuse (12 months), history of repeated minor head injury or a period of unconsciousness of 1 h or more, high consumption of caffeine (≥500 mg/d), marijuana use (in prior 2 months), tobacco use (in prior 6 months), an active infection, use of psychotropic medications (in prior 4 weeks), use of supplements known to improve cognitive function, an allergy to ingredients of the test product or the snack provided, or a sleep disorder or occupation where sleep during the overnight hours was irregular.

### Reaction performance assessments

**Fig. 3:**
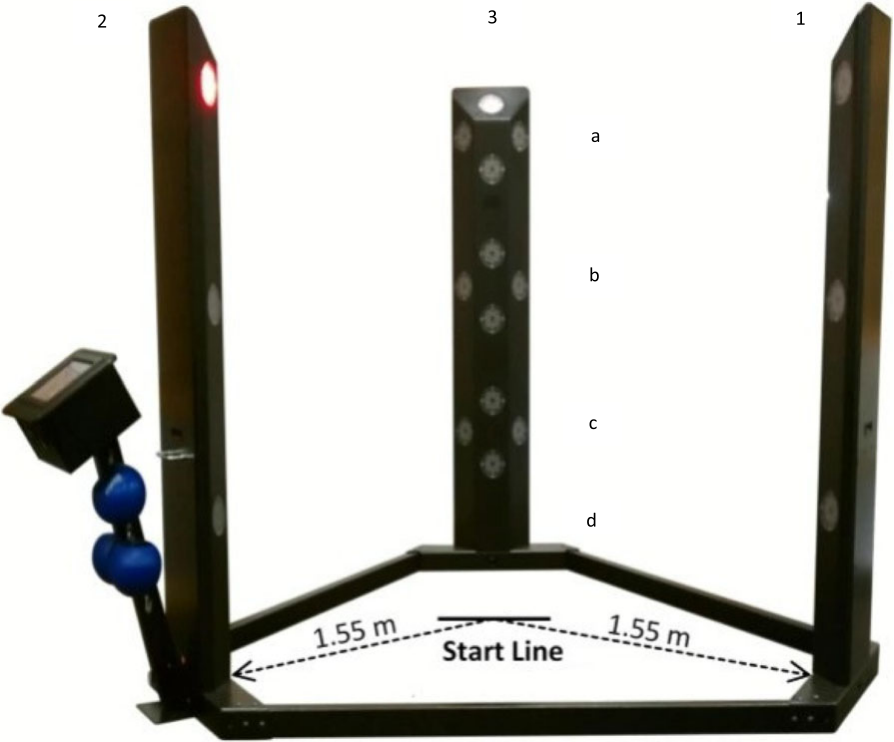
Makoto device used for measurement of reactive agility

**Fig. 4:**
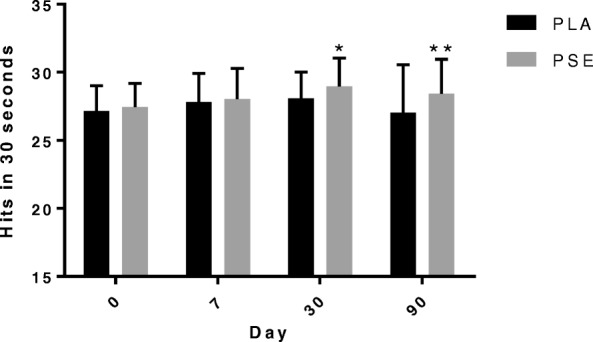
Number of hits obtained with the stationary Makoto with footplates assessment for 30 s. Abbreviations: PSE, proprietary spearmint extract; PLA, placebo. Data are presented as mean ± SD. * represents *p* < 0.05 difference from PLA; ** represents *p* < 0.01 difference from PLA

The interactive audio-visual reaction time test battery was performed on the Makoto Arena II device (Makoto USA Inc., Elk Grove Village, IL), a device which has been employed in studies previously [40, 44–47]. The Makoto device is a 360^0^ audio-visual device consisting of 3 towers 1.83 m tall in the shape of a triangle with 2.44 m between towers. Each tower consists of 16 targets: 12 on each tower (levels a, b, and c; see Fig. 3) and 4 on each footplate on the floor (level d; see Fig. 3) intended to be struck with hands and feet, respectively. A line of black tape was placed on the ground whose center is 1.55 m from each of the first two towers. Participants started behind the line and faced forward for all tests. Ten tests were utilized to address a variety of perception and movement modalities. The first three tests involved a single-step protocol, where a single target on one of two towers (tower 1 or 2, level b, top of the diamond of four panels; see Fig. 3) would light up once and the participant would move toward the target and strike it. The three single-step tests utilized either an audio stimulus, a visual stimulus, or a combined audio and visual stimulus. To eliminate guessing order by the participant, the single-step tests were each performed five times in a random order with two tests to the left (tower 3), two tests to the right (tower 1), and one random direction (left or right) with its score discarded. Duplicate scores for each side (left or right) were averaged for reporting purposes, such that each of the three single-step tests resulted in a score for left and a score for right. The remaining seven tests were all timed tests with the panels exhibiting both audio and visual stimuli in unison until the allotted time was completed. The fourth and fifth tests were 30 s stationary tests without or with footplates (level d), in which the participant was in front of one tower (tower 1) and the 12 tower targets (or 16 targets with footplates) were struck as quickly as possible. The sixth and seventh tests were lateral movement tests utilizing two towers (towers 1 and 2), which involved the participant moving back and forth between the two towers as the 12 hand targets from each of the towers (or 16 targets with footplates) activated one at a time for 30 s. The eighth and ninth tests were 30-s multi-directional movement tests using all three towers, and involved the participant moving among the three towers as the 12 hand targets from each of the towers (or 16 targets with footplates). The tenth test was identical to the ninth, except that it lasted 2 min. All the 30-s tests (tests 4–9) were performed in duplicate and scores were averaged. Reaction performance was captured as the number of hits and average reaction time (ART) for all tests, except the single step testing for which only one hit occurred each test and therefore only reaction time was captured. Targets were activated for a maximum of 10 s, so activated targets could not be missed since participants had ample time to strike them. Since the single step tests were short in duration and not fatiguing, the rest periods were as short as possible: 5 s. Between every iteration of each 30-s test, participants rested for 30 s. Prior to the 2-min test, participants were given 60 s of rest.

### Inflammatory biomarkers and safety assessments

After a 10 h overnight fast, blood samples were collected via venipuncture by a trained phlebotomist. After collection, samples were analyzed (Laboratory Corporation of America, Burlington, NC) for the following parameters: complete blood count (CBC) [white blood cell count (WBC), red blood cell count (RBC), hemoglobin, hematocrit, mean corpuscular volume (MCV), mean corpuscular hemoglobin (MCH), mean corpuscular hemoglobin concentration (MCHC), red blood cell distribution width (RDW), platelets (absolute), neutrophils (percent and absolute), lymphocytes (percent and absolute), monocytes (percent and absolute), eosinophils (percent and absolute), basophils (percent and absolute)], comprehensive metabolic panel (CMP) [serum glucose, blood urea nitrogen (BUN), creatinine, estimated glomerular filtration rate (eGFR), BUN:creatinine, sodium, potassium, chloride, carbon dioxide, calcium, protein, albumin, globulin, albumin:globulin (A/G), bilirubin, alkaline phosphatase, aspartate aminotransferase (AST), alanine aminotransferase (ALT)], lipids [total cholesterol, triglycerides, high density lipoprotein (HDL) cholesterol, very low density lipoprotein (VLDL), low density lipoprotein (LDL) cholesterol, LDL:HDL ratio], and biomarkers of stress and inflammation [cortisol, C-reactive protein, and interleukin 6 (IL6)]. Upon arrival to the laboratory and after resting seated for at least 5 min, vital signs (systolic and diastolic blood pressure, heart rate) were measured in duplicate via an automated digital sphygmomanometer (Omron M6 AC, Omron Inc., Kyoto, Japan) and averaged. Duplicate tests were conducted with a minimum of 2 min between tests. Blood pressure measurements were considered accurate if no more than 5 mmHg variation occurred between measurements. In addition to the above, participants were queried on adverse events (AE) throughout the trial.

### Exercise, sleep, and food logs

To control for changes in exercise, sleep, and diet, participants were asked to keep exercise, sleep, and diet constant throughout the study period. To monitor adherence, exercise, sleep, and food logs were submitted throughout the study. Participants were required to write down the hours and type of exercise performed daily such as strength, cardio, or a combined strength/cardio exercise (CrossFit, yoga, etc.). The exercise logs were checked each visit to ensure that participants did not exercise 24 h prior or exercise strenuously 48 h prior to study visits. The logs were also evaluated to confirm participants did not exercise more than 6 h/week or less than 1 h/week, the limits set by the inclusion criteria to which participants agreed to keep constant throughout the trial. Hours of sleep per night were also recorded. Average weekly hours of exercise and sleep were evaluated at each study visit. A pre-visit food log was captured during the 24 h prior to baseline (Day 0), photocopied and returned to the participants so that they could repeat this diet the day prior to each visit. A 3-day food log was recorded for three non-consecutive days (two weekdays and one weekend day) between each visit, so any changes in diet could be calculated.

### Statistical analyses

An evaluable sample size of 53 subjects per group was expected to provide 80% power (two-sided, α = 0.05) to detect an 8% difference in the number of hits in an assessment of reactive agility utilizing the Makoto device [44, 45]. A sample of at least 69 subjects per group was randomized to account for attrition and non-compliance (Fig. 1).

Statistical analyses were conducted using SAS version 9.2 (SAS Institute, Cary, NC) by an independent, third-party statistician (Summit Analytical, Denver, CO). Analyses were completed on an intent-to-treat (ITT) population which included all participants who were randomized into the study and consumed at least one dose of study product. A mixed model of repeated measures (MMRM) analysis was fit to the data for each continuous and assumed continuous study variable. All MMRM models contained the main effect of treatment (active vs. control), the main effect of time, and the interaction between treatment and time. *P* values using a model fit to raw data values containing treatment (active vs. control), time (Days 0, 7, 30, and 90), and the treatment x time interaction were evaluated. To account for observed differences at baseline for the inflammatory marker, CRP, a mixed-effects analysis of covariance (ANCOVA) model was utilized a priori. The ANCOVA model contained treatment (active vs. control), time (Days 7, 30, and 90), and the treatment x time interaction where participants’ baseline data for a given outcome (Day 0) was included as the covariate. A Fisher’s exact test was applied to demographics and attrition. Total AE and AE related to the study product were evaluated by Chi square analysis. Effect sizes are presented as Cohen’s d and interpreted as 0.2 = small, 0.5 = medium, 0.8 = large. Significance levels for each test, main effect and interaction were evaluated at alpha = 0.05. Data are represented as means ± standard deviation (SD).

## Results

### Demographics

One hundred and forty-two individuals were randomized, 69 into the PLA and 73 into the PSE groups. Fifty-four and fifty-two participants completed the trial in the PLA and PSE treatment groups, respectively. Demographics are shown in Table 1. Treatment groups were well-balanced in demographics for the baseline characteristics. Although exercise as self-reported on the New Zealand Physical Activity score was different at the screening visit (*p* = 0.01), comparison of the actual hours of exercise collected on study diaries between screening and baseline visits (Day 0) indicated no differences between groups in exercise.

**Table 1:** Baseline demographics

Parameter	Overall Population	PLA	PSE	*P* value^1^
Age (years), Mean ± SD	27.5 ± 7.9	27.9 ± 7.8	27.2 ± 8.0	0.60
Race (n, %)				0.11
White	122 (86%)	55 (80%)	67 (92%)	
Black or African American	11 (8%)	7 (10%)	4 (5%)	
Asian	9 (6%)	7 (10%)	2 (3%)	
Gender (n, %)				0.89
Male	98 (69%)	48 (70%)	50 (69%)	
Female	44 (31%)	21 (30%)	23 (31%)	
BMI (kg/m^2^), Mean ± SD	25.9 ± 3.2	25.8 ± 3.3	25.9 ± 3.2	0.86
Sleep (h)^a^, Mean ± SD	7.2 ± 0.8	7.3 ± 0.8	7.2 ± 0.7	0.82
Exercise (h)^a^, Mean ± SD	3.8 ± 2.0	3.8 ± 1.9	3.7 ± 2.0	0.66
New Zealand Physical Activity Questionnaire (h), Mean ± SD	4.7 ± 1.3	5.0 ± 1.2	4.44 ± 1.4	0.01

*Abbreviations*: *BMI* body mass index, *PSE* proprietary spearmint extract, *PLA* placebo, *SD* standard deviation

^1^*P* value for PLA versus PSE calculated via Fisher’s exact test

^a^Data obtained from study diary collected for 7 days between phone screening and baseline visit

### Compliance

Compliance was not significantly different at any timepoint. At Day 90, compliance was 95.4 and 96.8% in the PLA and PSE groups, respectively, with no difference between groups (*p* = 0.328). Attrition among all participants was 25.4%, and it was not significantly different between treatments or genders (*p* = 0.5048).

### Reaction performance assessments

**Fig. 5:**
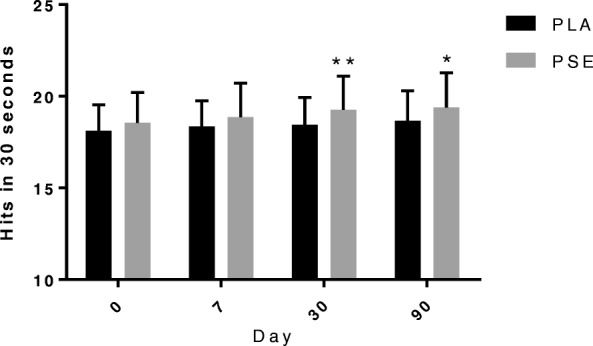
Hits obtained with the multi-directional Makoto with footplates assessment over 30 s. Abbreviations: PSE, proprietary spearmint extract; PLA, placebo. Data are presented as mean ± SD. * represents p < 0.05 difference from PLA; ** represents p < 0.01 difference from PLA

Data and statistical analyses for outcomes obtained utilizing the Makoto device are shown in Table 2. The hits obtained with the stationary test with footplates for 30 s (Fig. 4) were significantly higher in PSE compared with PLA at Day 30 (PSE vs. PLA: 28.96 ± 2.08 vs. 28.09 ± 1.92 hits; *p* = 0.040; d = 0.435) and Day 90 (PSE vs. PLA: 28.42 ± 2.54 vs. 27.02 ± 3.55 hits; *p* = 0.002; d = 0.454). ART for the stationary test with footplates measured for 30 s were significantly lower in PSE compared with PLA at Day 7 (PSE vs. PLA: 0.5896 ± 0.060 vs. 0.6141 ± 0.073 s; *p* = 0.049; d = − 0.367) and Day 30 (PSE vs. PLA: 0.5811 ± 0.068 vs. 0.6033 ± 0.055 s; p = 0.049; d = − 0.359). The hits obtained with the multi-directional test with footplates (Fig. 5) for 30 s were significantly higher in PSE compared with PLA at Day 30 (PSE vs. PLA: 19.25 ± 1.84 vs. 18.45 ± 1.48 hits; *p* = 0.007; d = 0.479) and Day 90 (PSE vs. PLA: 19.39 ± 1.90 vs. 18.66 ± 1.64 hits; *p* = 0.026; d = 0.411). Significant differences were not observed in the remaining Makoto tests.

**Table 2:** Data for Makoto performance outcomes

	Day 0	Day 7	Day 30	Day 90	*P* value
Single Step Audio Left (sec)					
PSE	1.0737 ± 0.118	1.0482 ± 0.135	1.0632 ± 0.131	1.0320 ± 0.121	0.129
PLA	1.1180 ± 0.144	1.0751 ± 0.113	1.0765 ± 0.120	1.0554 ± 0.108	
Single Step Audio Right (sec)					
PSE	1.0432 ± 0.112	1.0255 ± 0.157	1.0187 ± 0.116	1.0105 ± 0.118	0.081
PLA	1.0867 ± 0.136	1.0626 ± 0.113	1.0450 ± 0.123	1.0219 ± 0.101	
Single Step Visual Left (sec)					
PSE	0.9919 ± 0.163	0.9648 ± 0.146	0.9434 ± 0.132	0.9531 ± 0.134	0.305
PLA	1.0284 ± 0.158	0.9774 ± 0.127	0.9731 ± 0.116	0.9592 ± 0.118	
Single Step Visual Right (sec)					
PSE	0.9580 ± 0.140	0.9362 ± 0.179	0.9065 ± 0.130	0.9076 ± 0.126	0.304
PLA	0.9840 ± 0.151	0.9520 ± 0.107	0.9455 ± 0.118	0.9185 ± 0.122	
Single Step Left (sec)					
PSE	0.9577 ± 0.138	0.9215 ± 0.139	0.9309 ± 0.115	0.8935 ± 0.123	0.483
PLA	0.9770 ± 0.127	0.9553 ± 0.114	0.9266 ± 0.116	0.9012 ± 0.099	
Single Step Right (sec)					
PSE	0.9064 ± 0.128	0.9075 ± 0.122	0.8732 ± 0.116	0.8824 ± 0.104	0.306
PLA	0.9334 ± 0.127	0.9212 ± 0.102	0.8958 ± 0.114	0.8872 ± 0.116	
Stationary Hits (hits)					
PSE	28.90 ± 1.91	29.64 ± 2.73	30.75 ± 2.33	30.74 ± 2.38	0.481
PLA	28.93 ± 2.18	29.39 ± 2.29	30.15 ± 2.28	30.30 ± 2.21	
Stationary ART (sec)					
PSE	0.5681 ± 0.061	0.5384 ± 0.072	0.5256 ± 0.061	0.5209 ± 0.059	0.331
PLA	0.5789 ± 0.064	0.5509 ± 0.059	0.5347 ± 0.055	0.5290 ± 0.051	
Stationary wF Hits (hits)					
PSE	27.45 ± 1.75	28.04 ± 2.24	28.96 ± 2.08^*^	28.42 ± 2.54^**^	0.019
PLA	27.16 ± 1.84	27.81 ± 2.09	28.09 ± 1.92	27.02 ± 3.55	
Stationary wF ART (sec)					
PSE	0.6242 ± 0.069	0.5896 ± 0.060^*^	0.5811 ± 0.068^*^	0.5698 ± 0.057	0.036
PLA	0.6422 ± 0.066	0.6141 ± 0.073	0.6033 ± 0.055	0.5914 ± 0.053	
Lateral Hits (hits)					
PSE	21.81 ± 1.77	22.12 ± 2.32	22.60 ± 2.24	22.84 ± 2.26	0.187
PLA	21.45 ± 2.02	21.79 ± 1.80	22.07 ± 1.82	22.37 ± 1.81	
Lateral ART (sec)					
PSE	0.9171 ± 0.116	0.8859 ± 0.134	0.8856 ± 0.136	0.8616 ± 0.132	0.225
PLA	0.9516 ± 0.131	0.9179 ± 0.108	0.9022 ± 0.103	0.8840 ± 0.104	
Lateral wF Hits (hits)					
PSE	21.75 ± 1.73	22.22 ± 1.81	22.55 ± 1.88	22.72 ± 1.86	0.203
PLA	21.48 ± 1.79	21.88 ± 1.68	22.07 ± 1.51	22.42 ± 1.64	
Lateral wF ART (sec)					
PSE	0.9207 ± 0.124	0.8734 ± 0.103	0.8820 ± 0.116	0.8588 ± 0.116	0.150
PLA	0.9453 ± 0.109	0.9141 ± 0.113	0.9019 ± 0.086	0.8768 ± 0.088	
Multi-directional Hits (hits)					
PSE	18.95 ± 1.41	19.14 ± 1.66	19.60 ± 1.59	19.65 ± 1.56	0.143
PLA	18.76 ± 1.49	18.87 ± 1.38	19.04 ± 1.35	19.26 ± 1.34	
Multi-directional ART (sec)					
PSE	1.1276 ± 0.131	1.0979 ± 0.130	1.0908 ± 0.129^*^	1.0730 ± 0.131	0.079
PLA	1.1641 ± 0.117	1.1351 ± 0.113	1.1288 ± 0.106	1.0983 ± 0.098	
Multi-directional wF Hits (hits)					
PSE	18.54 ± 1.66	18.86 ± 1.86	19.25 ± 1.84^**^	19.39 ± 1.90^*^	0.020
PLA	18.12 ± 1.41	18.34 ± 1.41	18.45 ± 1.48	18.66 ± 1.64	
Multi-directional wF ART (sec)					
PSE	1.1684 ± 0.157	1.1303 ± 0.162^*^	1.1228 ± 0.160^*^	1.1025 ± 0.155	0.051
PLA	1.2058 ± 0.139	1.1825 ± 0.132	1.1788 ± 0.124	1.1550 ± 0.140	
Multi-directional wF Hits 2 min (hits)					
PSE	71.5 ± 6.7	72.4 ± 7.1	74.8 ± 7.5	75.2 ± 7.4	0.178
PLA	70.1 ± 5.3	71.6 ± 5.7	72.1 ± 5.7	73.0 ± 6.1	
Multi-directional wF ART 2 min (sec)					
PSE	1.188 ± 0.171	1.152 ± 0.169	1.133 ± 0.158	1.110 ± 0.154	0.242
PLA	1.222 ± 0.136	1.181 ± 0.135	1.176 ± 0.124	1.160 ± 0.142	

*Abbreviations*: *PSE* proprietary spearmint extract, *PLA* placebo, *wF* with footplates, *ART* average reaction time, *SD* standard deviation

Data are presented as mean ± SD. Treatment *P* values are shown. ^*^represents *p* < 0.05 difference from PLA; ^**^ represents *p* < 0.01 difference from PLA

### Biomarkers of stress and inflammation

**Fig. 6:**
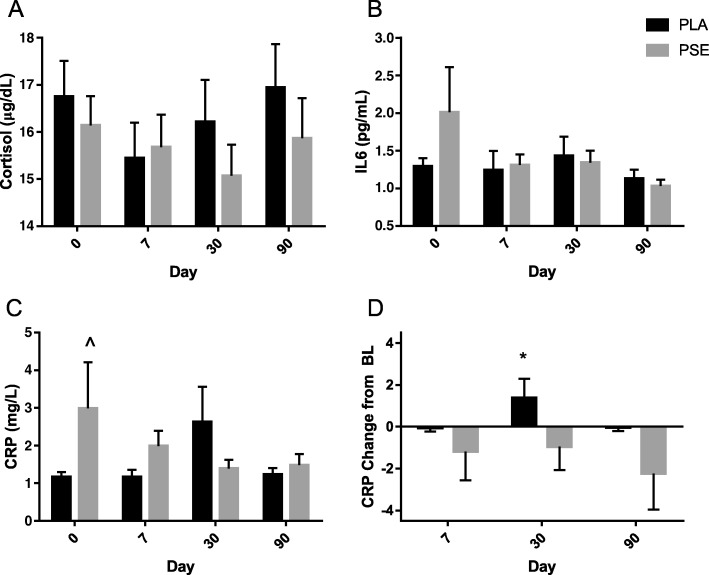
Stress and Inflammatory Markers. Cortisol (**a**), interleukin 6 (IL6, **b**), C-Reactive Protein (CRP) raw scores (**c**) and CRP change from baseline (**d**) are shown. Abbreviations: PSE, proprietary spearmint extract; PLA, placebo; BL, baseline. Data are presented as mean ± SD. ^ indicates *p* = 0.030 difference at baseline, therefore ANCOVA was utilized to identify a significant treatment x visit interaction with between group differences at Day 30 (* *p* < 0.05)

Plasma cortisol, IL6 and CRP were measured at all timepoints and are shown in Fig. 6. Comparisons between PSE and PLA were not significantly different for cortisol and IL6. Differences at baseline (*p* = 0.03) were present for CRP; therefore, ANCOVA was utilized to identify a significant treatment x visit interaction (p = 0.049) and pairwise comparisons revealed that PSE significantly decreased CRP at Day 30 compared to PLA (*p* = 0.045).

### Safety assessment

Adverse events were collected for all participants throughout the study. There was no difference in the total number of AE between groups (*p* = 0.5935). In addition, the total number of AE related to study product did not differ between groups (*p* = 0.3020). Complete blood count, complete metabolic profile, lipids, body metrics, and vital signs were assessed to evaluate overall health status over the study period. No overall treatment effects were observed for any of the outcomes (Additional file 1: Tables S1-S4). Four outcomes: absolute monocytes, absolute granulocytes, granulocyte percentages, and systolic blood pressure did evince significant treatment x visit interactions; however, any differences evident at day 30 were not significant at day 90 and all values remained within normal limits and were not deemed clinically relevant.

### Diet, exercise, and sleep measurements

Evaluation of diet records collected at each study period indicated no difference between groups in total calorie, protein, fat, or carbohydrate intake (Table 3). Participants appeared to have maintained consistent diet over the 90-day study period and self-reported sleep and exercise also appeared to have been kept constant and did not differ between groups (Table 4).

**Table 3:** Self-reported diet evaluation

Diet	PLA Mean ± SD	PSE Mean ± SD	*P*-value
Calories (kcal)			
Day 0	2056 ± 604.6	2063 ± 603.7	0.545
Day 7	2103 ± 492.6	2029 ± 598.5	
Day 30	2028 ± 599.4	1997 ± 572.9	
Day 90	2074 ± 655.7	1952 ± 611.2	
Protein (g)			
Day 0	125.30 ± 45.8	125.05 ± 59.5	0.818
Day 7	126.01 ± 46.6	126.05 ± 60.1	
Day 30	126.05 ± 53.6	128.33 ± 66.0	
Day 90	128.17 ± 56.3	122.52 ± 57.1	
Fat (g)			
Day 0	79.14 ± 34.4	74.98 ± 22.5	0.354
Day 7	87.24 ± 40.0	82.22 ± 39.1	
Day 30	75.63 ± 30.9	73.80 ± 25.0	
Day 90	74.97 ± 29.5	71.71 ± 35.0	
Carbohydrate (g)			
Day 0	212.61 ± 81.0	218.77 ± 78.9	0.519
Day 7	221.88 ± 82.4	203.71 ± 78.3	
Day 30	214.78 ± 81.1	204.83 ± 64.6	
Day 90	218.88 ± 81.9	201.48 ± 75.3	

*Abbreviations*: *SD* standard deviation, *PSE* spearmint, *PLA* placebo

Treatment *P* values are shown

**Table 4:** Self-reported sleep and exercise

Sleep and exercise	PLA Mean ± SD	PSE Mean ± SD	*P*-value
Self-Reported Sleep (h)			
Day 0	7.26 ± 0.81	7.23 ± 0.74	0.223
Day 7	7.40 ± 0.79	7.01 ± 0.81	
Day 30	7.33 ± 0.91	7.08 ± 0.89	
Day 90	7.30 ± 0.73	7.16 ± 0.72	
Self-Reported Exercise (min)			
Day 0	230.77 ± 115.8	222.81 ± 122.6	0.761
Day 7	236.88 ± 134.3	236.57 ± 133.4	
Day 30	226.47 ± 113.6	234.52 ± 132.1	
Day 90	234.61 ± 105.7	221.68 ± 152.4	

*Abbreviations*: *SD* standard deviation, *PSE* proprietary spearmint extract, *PLA* placebo

Treatment *P* values are shown

## Discussion

The results of the present study suggest that consumption of PSE can improve reactive agility. We formally accept our hypothesis that daily supplementation of 900 mg of PSE can significantly improve selected markers of reactive agility, specifically stationary and multi-directional when both hand and foot strikes are involved. The multi-directional test with footplates evinced improvements in hits after long-term supplementation (Day 30 and Day 90), and the reaction time trended toward improvement. The number of hits on the stationary test with footplates was significantly higher after long-term supplementation (Day 30 and Day 90), and the reaction time improved at Day 7 and Day 30, with a trend observed at Day 90.

To our knowledge, there are no existing studies on reactive agility after supplementation with botanical ingredients. The Makoto testing utilized in the present study may address the cognitive aspects of reactive agility, such as choice reaction time, more than the physical aspects, when compared to more common reactive agility testing [41, 48]. A typical reactive agility test consists of unidirectional running with one unplanned change-of-direction [41], which relies more on physical aspects of agility than the Makoto testing, which utilizes multiple stimuli to invoke constant changes of direction, speed, and decision making and may favor the cognitive aspects, such as response time. Therefore, it may be useful to compare the present findings to cognitive studies that utilized choice reaction time testing. Results from the present study are supported by existing literature on choice reaction time in botanical extracts from the mint family containing rosmarinic acid. Acute administration of 333 mg of an ethanolic extract of sage was found to improve choice reaction time at 4 and 6 h [14]. In addition, acute administration of doses of 300–900 mg of a methanolic lemon balm improved choice reaction time at 2.5, 4 and 6 h post-supplementation [49]. A second sage study demonstrated no significant changes in choice reaction time; however, the extract was an essential oil high in monoterpenoids and low in rosmarinic acid, which could account for the difference in findings [50]. While the previous studies used acute supplementation of either solvent extracted extracts of the mint family or an essential oil, the present study involved chronic administration of a natural water-extracted PSE. In addition, the choice reaction time testing utilized in the previous studies was very simple (involved pressing “YES” or “NO” buttons when these words were presented on the screen) and passive, while the present study measured active choice reaction performance with tests that varied in complexity, such as the addition of footplates.

Though few studies have compared active and passive choice reaction performance testing, data from our laboratory suggest that results may vary between the two types of tests. In one study, healthy, active participants were dehydrated and choice reaction performance was compared pre and post-dehydration utilizing both active testing via Makoto and passive choice reaction performance via Stroop test and shifting attention test [40]. Surprisingly, dehydration improved choice reaction time as assessed in the shifting attention test and decreased errors during the Stroop test; however, active choice reaction performance significantly decreased via 30 s multi-directional test without footplates as measured with Makoto. The findings demonstrate that active and passive choice reaction testing may yield significantly different results, suggesting that active testing may be more relevant in athletic populations where improved exercise performance may be the goal. Therefore, the use of a novel testing device, such as the Makoto Arena II, which can measure choice reaction time while the participant is active, is important to elucidate the connection between cognitive and physical activity.

In the current study, the tests in which significant differences were evident, the stationary and multi-directional tests, also involved footplates. The inclusion of footplates presented an additional shifting attention component, as the participant was required to switch between responding with hands or feet. While classic cognitive attention tests do not require the participants to make similar attention shifts, one study on sage and two studies on PSE evaluated attention and generally support the findings of the present study. An acute dose of 333 mg of sage was shown to induce improvements in both accuracy and speed of attention [14]. An open-label study on older individuals with self-reported memory impairment supplemented with 900 mg of PSE improved attention at 2 and 4 h after acute supplementation and after 30 days of chronic supplementation [34]. Additionally, a small-scale study in younger participants numerically improved attention after acute administration with 900 mg of PSE [36]. Furthermore, the addition of footplates may not target attention (sensory input) as much as it highlights a complex psychomotor task (output). Unfortunately, no previous studies could be found that investigated shifting hand and foot responses. Of the studies that have previously utilized the Makoto device, two did not involve footplates [44, 45]. Studies from our laboratory have utilized the Makoto Arena II with footplates; however, neither study directly compared tests with and without footplates [40, 46]. One study utilized tests with and without footplates [40], but the tests varied to such a degree that direct comparisons could not be made. Another study demonstrated sex differences in reaction time after dehydration, but only the multi-directional, 2-min test with footplates was utilized [46]. Since the tests on the Makoto are physical performance tasks, there are important differences in the demands on the lower body when footplates are added from a motor and psychomotor perspective. To our knowledge, the present study is the first to demonstrate improvements when the subjects were required to shift attention between hand and foot strikes.

The ability to rapidly shift attention between hand and foot strikes could have practical relevance to sport-specific athletic performance. One relevant application would be in combat sports that allow hand and foot strikes, such as mixed martial arts or kickboxing. Some of the other sports where shifting attention between hands and feet might affect performance include any sport that requires feet to be rapidly adjusted to a set stance and then hands are primarily utilized. Some examples include: baseball (running for a ball, then catching and throwing), basketball (running to a shooting spot without the ball, then catching, passing, and shooting), volleyball or tennis (running to a spot, then setting feet and hitting the ball). Though athletic performance and exercise performance were not tested directly, active choice reaction performance testing will better translate to athletic performance than standard, passive testing (i.e. cognitive tests that do not involve complex physical responses), and future studies should investigate whether the hypothesis that the beneficial effects that were observed in the present study will translate to factual improvements in athletic performance in response to PSE supplementation.

Several of the results on certain reactive agility tests did not achieve significance, most likely due to variations in the specific domains targeted by the individual tests. The three types of tests where results were not significant were the lateral test, the single-step tests, and the multi-directional test for 2 min. The lateral test is a sports-specific movement which is highly practiced among specific elite athletes. An example would be when facing an opponent, such as football, soccer, or wrestling. The general population does not practice lateral movements and most likely would not be very proficient, potentially leading to wider variability and less sensitivity in the measurements. In contrast, the stationary test (in which we identified differences following PSE supplementation in the current study) does not require movement among towers; therefore, it does not require proficiency in any sport-related movement and could be better suited for a recreationally active population as used in the current study. The multi-directional test (another test where differences were identified in the current study), while involving lower body movement, does not require movement that is as specific as the lateral test. The spinning and pivoting movements that are needed to respond to the 3 towers in the multi-directional test are movements that are required in everyday life to respond to stimuli in 360^0^ of space, whereas the lateral movement in the lateral test occurs less frequently in quotidian life. Therefore, the multi-directional test may be better suited than the lateral test for the recreationally active population used in the current study. The single-step tests, while not requiring any sports-specific proficiency, do involve a skill that may also be better suited to a population of elite athletes. The single step tests measure the initial burst of acceleration or “first step” after a stimulus is presented; however, acceleration may not be as crucial for recreational athletes and, therefore, not be sensitive enough to detect changes following PSE consumption in this population. The multi-directional test when administered for 2 min is designed to incorporate the influence of fatigue. The results for the multi-directional test with footplates for 30 s were significantly improved following PSE supplementation, thus demonstrating choice reaction time performance in a 360^0^ audio and visual field with a shifting attention component was improved. However, when conducting the identical test elongated for a total of 2 min, fatigue becomes a dominant factor as reaction times become slower. Therefore, the lack of significance in this longer test suggests that the current study may not have been powered to evaluate the confounding factor of fatigue on performance. Finally, since the results of the tests without footplates were not significant, this suggests that PSE consumption significantly enhances performance only in more multi-dimensional, more complex tests of reactive agility.

Blood biomarkers of inflammation and stress were evaluated with all outcomes showing improvement over baseline; however, only CRP scores were significant. The decrease in the inflammatory biomarker CRP is congruent with traditional usage of mint as an herbal medicine [17] and the literature in which spearmint decreased inflammatory signaling. Though studies in humans are scarce, multiple in vitro and in vivo studies have demonstrated the anti-inflammatory benefits of spearmint consumption [51–53]. A variety of spearmint bred for high rosmarinic acid was incubated in gastric and intestinal fluids to simulate digestion and absorption, then subsequently exposed to cartilage cultured with lipopolysaccharide (LPS) [51]. The spearmint significantly inhibited LPS-induced prostaglandin E2 and nitric oxide expression. In rats, four solvent fractions of *Mentha spicata* were evaluated after acute and 7-day chronic oral administration [53]. The ethyl acetate extract (160 mg/kg body weight in 25% DMSO) reduced inflammation after both acute and chronic administration at levels comparable to anti-inflammatory drugs, which served as a positive control. Pearson et al. demonstrated that horses fed 28.1 g/d of high-rosmarinic acid mint for 24 days and then injected with LPS on day 21 exhibited reduced markers of inflammation and immune response in their synovial fluid, including prostaglandin E2, glycosaminoglycans, white blood cells, segmented neutrophils, and lymphocytes [52].

Although the PSE in the current study is water-extracted, the polyphenolic components may provide anti-inflammatory benefits that could contribute to the observed improvements in reactive agility. Inflammation plays a major role in brain function [54], and CRP has been shown to be directly neurotoxic [55]. In neurodegenerative disorders, brain cells produce CRP and other complement proteins leading to chronic inflammation and potentially neuronal cell death [56, 57]. Direct reduction of CRP may be neuroprotective; therefore, the present data suggest that chronic administration of PSE and the resulting improvements on CRP may indirectly contribute to improvements in choice reaction performance via reduction of the low-level inflammation in young, healthy individuals.

The results from the safety blood panel demonstrated that supplementation with PSE for 90 days does not negatively affect complete blood count, complete metabolic profile, blood lipids, or vital signs. PSE has been shown to be well tolerated in previous studies and the current trial expands upon the body of evidence supporting safety [34–36].

There are limitations regarding interpretation of the results in the current study. First, data collection occurred over a period of several months, which meant that the same laboratory personnel were not able to conduct all the Makoto testing. Therefore, variability in results may have increased due to multiple testing administrators and some variation in their testing approach. However, to minimize variability, all testing administrators were trained by the same person and using the same exact protocols. Second, within subject motivation can be challenging to maintain, especially when testing visits occur months apart, and can affect test scores. To keep participants motivated, researchers utilized verbal cues to keep participants engaged and encouraged. Future studies could involve some subjective measurement of motivation to ensure consistency across visits. Third, even though a familiarization visit was utilized as recommended by previous reliability studies [47], learning effects could have occurred throughout the study. Learning effects should have been consistent amongst groups; therefore, comparison to a placebo group should have negated the impact of the learning effects.

## Conclusion

The present study is the first to observe an improvement in reactive agility following consumption of 900 mg of PSE for 90 days, as measured by the Makoto device using both the stationary test and the multi-directional test with footplates at selected timepoints. By demonstrating improvements on specific measurements of choice reaction performance in a young, healthy population, the findings of the present study expand on previous studies on PSE which showed improvement in cognitive function. The present study supports the use of PSE as a safe nootropic with potential downstream benefits in athletic performance, particularly in combat sports. Future research should be conducted to verify the hypothesis that the measured improvements in reactive agility will indeed translate to statistically significant and practically relevant improvements in athletic performance.

## Additional file

Additional file 1: Supplemental Safety Tables. (DOCX 82.6 kb)

## Abbreviations

A/G: Albumin:Globulin
AE: Adverse event
ALT: Alanine aminotransferase
ANCOVA: Analysis of covariance
ART: Average reaction time
AST: Aspartate aminotransferase
BUN: Blood urea nitrogen
CBC: Complete blood count
CMP: Comprehensive metabolic panel
eGFR: Estimated glomerular filtration rate
HDL: High density lipoprotein
IL6: Interleukin 6
LDL: Low density lipoprotein
LPS: Lipopolysaccharide
MCH: Mean corpuscular hemoglobin
MCHC: Mean corpuscular hemoglobin concentration
MCV: Mean corpuscular volume
MMRM: Mixed model of repeated measures
PLA: Placebo
PSE: Proprietary spearmint extract
RBC: Red blood cell count
RDW: Red blood cell distribution
SD: Standard deviation
VLDL: Very low density lipoprotein
WBC: White blood cell count
wF: with footplates
