# Supplementary material for: Efficacy of a nootropic spearmint extract on reactive agility: a randomized, double-blind, placebo-controlled, parallel trial
Source: J Int Soc Sports Nutr. 2018 Dec 12;15:58. doi: 10.1186/s12970-018-0264-5 (PMC6291964; doi:10.1186/s12970-018-0264-5)
Supplement: Supplementary file 1 — Supplemental Safety Tables. (DOCX 82.6 kb) [file 12970_2018_264_MOESM1_ESM.docx]

Supplemental Table S1. Complete Blood Count.

| **Qu Outcome** | **PLA**  **Mean ± SD** | **PSE**  **Mean ± SD** | **P-value** |
| --- | --- | --- | --- |
| White Blood Cells (10^3^/µl) |  |  |  |
| Day 0 | 5.97 ± 1.60 | 5.94 ± 1.53 | 0.646 |
| Day 7 | 5.80 ± 1.60 | 5.88 ± 1.79 |  |
| Day 30 | 5.94 ± 1.78 | 5.69 ± 1.40 |  |
| Day 90 | 5.70 ± 1.48 | 5.69 ± 1.39 |  |
| Red Blood Cells (10^3^/µl) |  |  |  |
| Day 0 | 5.10 ± 0.43 | 5.15 ± 0.41 | 0.294 |
| Day 7 | 5.03 ± 0.42 | 5.13 ± 0.62 |  |
| Day 30 | 5.04 ± 0.43 | 5.10 ± 0.41 |  |
| Day 90 | 5.03 ± 0.43 | 5.15 ± 0.38 |  |
| Hemoglobin (g/dl) |  |  |  |
| Day 0 | 15.57 ± 1.32 | 15.73 ± 1.17 | 0.326 |
| Day 7 | 15.29 ± 1.35 | 15.52 ± 1.15 |  |
| Day 30 | 15.36 ± 1.52 | 15.61 ± 1.06 |  |
| Day 90 | 15.29 ± 1.38 | 15.73 ± 1.05 |  |
| Hematocrit (%) |  |  |  |
| Day 0 | 46.17 ± 3.44 | 46.56 ± 3.08 | 0.334 |
| Day 7 | 45.50 ± 3.37 | 46.00 ± 3.01 |  |
| Day 30 | 45.79 ± 3.59 | 46.63 ± 2.83 |  |
| Day 90 | 45.45 ± 3.47 | 46.76 ± 2.84 |  |
| Mean Corpuscular Volume (fL) | |  |  |
| Day 0 | 90.80 ± 3.73 | 90.50 ± 3.99 | 0.628 |
| Day 7 | 90.60 ± 3.78 | 90.70 ± 4.02 |  |
| Day 30 | 91.00 ± 3.45 | 91.20 ± 4.19 |  |
| Day 90 | 90.70 ± 3.49 | 91.00 ± 3.46 |  |
| Mean Corpuscular Hemoglobin (pg) | |  |  |
| Day 0 | 30.58 ± 1.34 | 30.58 ± 1.49 | 0.922 |
| Day 7 | 30.44 ± 1.47 | 30.58 ± 1.49 |  |
| Day 30 | 30.47 ± 1.48 | 30.68 ± 1.45 |  |
| Day 90 | 30.45 ± 1.33 | 30.60 ± 1.33 |  |
| Mean Corpuscular Hemoglobin Concentration (g/dL) | | |  |
| Day 0 | 33.71 ± 0.801 | 33.78 ± 0.743 | 0.421 |
| Day 7 | 33.59 ± 0.830 | 33.73 ± 0.650 |  |
| Day 30 | 33.51 ± 0.952 | 33.65 ± 0.652 |  |
| Day 90 | 33.62 ± 0.775 | 33.63 ± 0.636 |  |
| Red Blood Cell Distribution Width (%) | |  |  |
| Day 0 | 13.42 ± 0.667 | 13.47 ± 0.677 | 0.572 |
| Day 7 | 13.44 ± 0.692 | 13.50 ± 0.646 |  |
| Day 30 | 13.49 ± 0.656 | 13.38 ± 0.517 |  |
| Day 90 | 13.39 ± 0.637 | 13.46 ± 0.536 |  |
| Platelets (10^3^/µl) |  |  |  |
| Day 0 | 249.3 ± 48.6 | 251.3 ± 53.8 | 0.872 |
| Day 7 | 250.0 ± 45.8 | 250.1 ± 55.5 |  |
| Day 30 | 251.8 ± 52.7 | 246.5 ± 61.4 |  |
| Day 90 | 255.8 ± 55.6 | 243.4 ± 56.1 |  |
| Neutrophils (%) |  |  |  |
| Day 0 | 53.70 ± 9.59 | 51.30 ± 9.02 | 0.127 |
| Day 7 | 51.80 ± 8.95 | 50.50 ± 10.35 |  |
| Day 30 | 52.30 ± 8.77 | 51.80 ± 8.16 |  |
| Day 90 | 54.10 ± 9.53 | 51.00 ± 8.39 |  |
| Lymphocytes (%) |  |  |  |
| Day 0 | 34.50 ± 8.07 | 36.20 ± 8.18 | 0.302 |
| Day 7 | 36.70 ± 9.54 | 37.00 ± 8.41 |  |
| Day 30 | 35.80 ± 8.68 | 35.70 ± 7.27 |  |
| Day 90 | 34.70 ± 8.23 | 36.60 ± 7.73 |  |
| Monocytes (%) |  |  |  |
| Day 0 | 8.20 ± 2.02 | 8.60 ± 1.86 | 0.623 |
| Day 7 | 8.40 ± 3.84 | 8.20 ± 1.94 |  |
| Day 30 | 8.30 ± 1.89 | 8.40 ± 1.91 |  |
| Day 90 | 7.60 ± 1.87 | 8.50 ± 1.82 |  |
| Eosinophils (%) |  |  |  |
| Day 0 | 3.10 ± 2.92 | 3.50 ± 2.48 | 0.258 |
| Day 7 | 3.20 ± 2.84 | 3.70 ± 2.37 |  |
| Day 30 | 3.00 ± 2.22 | 3.50 ± 2.77 |  |
| Day 90 | 3.10 ± 2.45 | 3.40 ± 2.06 |  |
| Basophils (%) |  |  |  |
| Day 0 | 0.50 ± 0.50 | 0.50 ± 0.53 | 0.635 |
| Day 7 | 0.60 ± 0.54 | 0.70 ± 0.64 |  |
| Day 30 | 0.50 ± 0.50 | 0.50 ± 0.54 |  |
| Day 90 | 0.50 ± 0.50 | 0.40 ± 0.50 |  |
| Neutrophils Absolute (10^3^/µl) | |  |  |
| Day 0 | 3.31 ± 1.27 | 3.10 ± 1.19 | 0.291 |
| Day 7 | 3.08 ± 1.36 | 3.07 ± 1.46 |  |
| Day 30 | 3.20 ± 1.41 | 2.99 ± 1.06 |  |
| Day 90 | 3.15 ± 1.23 | 2.96 ± 1.05 |  |
| Lymphocytes Absolute (10^3^/µl) | |  |  |
| Day 0 | 2.02 ± 0.60 | 2.08 ± 0.59 | 0.641 |
| Day 7 | 2.05 ± 0.61 | 2.08 ± 0.50 |  |
| Day 30 | 2.05 ± 0.57 | 1.98 ± 0.43 |  |
| Day 90 | 1.93 ± 0.51 | 2.02 ± 0.44 |  |
| Monocytes Absolute (10^3^/µl) | |  |  |
| Day 0 | 0.48 ± 0.13 | 0.51 ± 0.17 | 0.691^1^ |
| Day 7 | 0.45 ± 0.13 | 0.48 ± 0.17 |  |
| Day 30 | 0.49 ± 0.18 | 0.47 ± 0.14 |  |
| Day 90 | 0.44 ± 0.14 | 0.49 ± 0.17 |  |
| Eosinophils Absolute (10^3^/µl) | |  |  |
| Day 0 | 0.19 ± 0.19 | 0.21 ± 0.17 | 0.181 |
| Day 7 | 0.19 ± 0.17 | 0.22 ± 0.17 |  |
| Day 30 | 0.17 ± 0.12 | 0.21 ± 0.20 |  |
| Day 90 | 0.17 ± 0.14 | 0.23 ± 0.20 |  |
| Basophils Absolute (10^3^/µl) |  |  |  |
| Day 0 | 0.01 ± 0.03 | 0.01 ± 0.04 |  |
| Day 7 | 0.02 ± 0.04 | 0.02 ± 0.04 | 0.594 |
| Day 30 | 0.01 ± 0.03 | 0.01 ± 0.03 |  |
| Day 90 | 0.01 ± 0.02 | 0.01 ± 0.03 |  |
| Immature Granulocytes (%) |  |  |  |
| Day 0 | 0.0 ± 0.0 | 0.0 ± 0.0 |  |
| Day 7 | 0.0 ± 0.0 | 0.0 ± 0.0 | 0.058^2^ |
| Day 30 | 0.1 ± 0.2 | 0.0 ± 0.0 |  |
| Day 90 | 0.0 ± 0.0 | 0.0 ± 0.0 |  |
| Immature Granulocytes Absolute (10^3^/µl) | |  |  |
| Day 0 | 0.00 ± 0.0 | 0.00 ± 0.0 | 0.058^3^ |
| Day 7 | 0.00 ± 0.0 | 0.00 ± 0.0 |  |
| Day 30 | 0.01 ± 0.2 | 0.00 ± 0.0 |  |
| Day 90 | 0.00 ± 0.0 | 0.00 ± 0.0 |  |

Abbreviations: SD: standard deviation; PSE: proprietary spearmint extract; PLA: placebo. Treatment P values are shown. ^1^Treatment x visit interaction, p=0.028. ^2^Treatment x visit interaction, p=0.015. ^3^Treatment x visit interaction, p=0.015.

Supplemental Table S2. Comprehensive Metabolic Panel.

| **Qu Outcome** | **PLA**  **Mean ±** **SD** | **PSE**  **Mean ±** **SD** | **P-value** |
| --- | --- | --- | --- |
| Glucose (mg/dL) |  |  |  |
| Day 0 | 88.9 ± 7.6 | 91.1 ± 8.8 | 0.537 |
| Day 7 | 89.3 ± 7.3 | 90.6 ± 7.1 |  |
| Day 30 | 91.8 ± 19.5 | 92.4 ± 8.5 |  |
| Day 90 | 90.4 ± 7.7 | 92.2 ± 11.9 |  |
| Blood Urea Nitrogen (mg/dL) | |  |  |
| Day 0 | 15.4 ± 3.9 | 16.5 ± 4.6 | 0.192 |
| Day 7 | 15.4 ± 4.4 | 16.2 ± 4.2 |  |
| Day 30 | 15.6 ± 4.6 | 15.6 ± 4.6 |  |
| Day 90 | 15.4 ± 5.1 | 16.5 ± 4.0 |  |
| Creatinine (mg/dL) |  |  |  |
| Day 0 | 1.002 ± 0.20 | 0.995 ± 0.18 | 0.801 |
| Day 7 | 0.996 ± 0.18 | 1.008 ± 0.19 |  |
| Day 30 | 1.024 ± 0.18 | 0.981 ± 0.18 |  |
| Day 90 | 0.993 ± 0.17 | 1.008 ± 0.19 |  |
| Estimated Glomerular Filtration Rate (non-African American (ml/min/1.73 m^2^) | | |  |
| Day 0 | 96.0 ± 15.5 | 98.5 ± 17.7 | 0.430 |
| Day 7 | 96.4 ± 17.1 | 96.7 ± 19.4 |  |
| Day 30 | 92.3 ± 13.5 | 98.6 ± 18.3 |  |
| Day 90 | 94.9 ± 14.3 | 95.5 ± 17.8 |  |
| Estimated Glomerular Filtration Rate African American (ml/min/1.73 m^2^) | | |  |
| Day 0 | 114.0 ± 25.2 | 93.0 ± 6.4 | 0.350 |
| Day 7 | 110.7 ± 28.3 | 96.0 ± 16.5 |  |
| Day 30 | 102.5 ± 27.3 | 104.0 ± 10.2 |  |
| Day 90 | 111.0 ± 25.0 | 98.0 (+) |  |
| Blood Urea Nitrogen:Creatinine | |  |  |
| Day 0 | 15.6 ± 3.8 | 16.8 ± 4.5 | 0.123 |
| Day 7 | 15.7 ± 4.0 | 16.3 ± 4.3 |  |
| Day 30 | 15.5 ± 4.5 | 15.9 ± 3.9 |  |
| Day 90 | 15.6 ± 3.2 | 16.4 ± 4.5 |  |
| Sodium (mmol/L) |  |  |  |
| Day 0 | 140.3 ± 2.1 | 140.4 ± 1.9 | 0.659 |
| Day 7 | 140.8 ± 2.0 | 140.3 ± 1.9 |  |
| Day 30 | 140.5 ± 2.1 | 140.4 ± 1.9 |  |
| Day 90 | 140.7 ± 1.6 | 140.8 ± 1.9 |  |
| Potassium (mmol/L) |  |  |  |
| Day 0 | 4.41 ± 0.32 | 4.47 ± 0.32 | 0.284 |
| Day 7 | 4.49 ± 0.26 | 4.49 ± 0.32 |  |
| Day 30 | 4.43 ± 0.35 | 4.46 ± 0.37 |  |
| Day 90 | 4.33 ± 0.30 | 4.46 ± 0.34 |  |
| Chloride (mmol/L) |  |  |  |
| Day 0 | 101.4 ± 2.0 | 101.3 ± 1.9 | 0.937 |
| Day 7 | 102.1 ± 2.0 | 101.7 ± 2.2 |  |
| Day 30 | 101.7 ± 2.0 | 102.0 ± 2.3 |  |
| Day 90 | 101.8 ± 2.0 | 101.8 ± 2.2 |  |
| Total Carbon Dioxide (mmol/L) | |  |  |
| Day 0 | 22.5 ± 1.7 | 22.4 ± 1.6 | 0.566 |
| Day 7 | 22.5 ± 2.0 | 22.5 ± 1.7 |  |
| Day 30 | 22.5 ± 1.6 | 22.2 ± 1.7 |  |
| Day 90 | 21.9 ± 1.6 | 22.0 ± 1.5 |  |
| Calcium (mg/dL) |  |  |  |
| Day 0 | 9.46 ± 0.31 | 9.51 ± 0.30 | 0.391 |
| Day 7 | 9.42 ± 0.31 | 9.45 ± 0.39 |  |
| Day 30 | 9.38 ± 0.30 | 9.42 ± 0.35 |  |
| Day 90 | 9.40 ± 0.29 | 9.45 ± 0.31 |  |
| Total Serum Protein (g/dL) |  |  |  |
| Day 0 | 6.95 ± 0.37 | 7.04 ± 0.34 | 0.536 |
| Day 7 | 6.89 ± 0.39 | 6.91 ± 0.30 |  |
| Day 30 | 6.90 ± 0.37 | 6.90 ± 0.32 |  |
| Day 90 | 6.98 ± 0.35 | 6.96 ± 0.34 |  |
| Albumin (g/dL) |  |  |  |
| Day 0 | 4.57 ± 0.26 | 4.61 ± 0.23 | 0.131 |
| Day 7 | 4.50 ± 0.23 | 4.55 ± 0.24 |  |
| Day 30 | 4.46 ± 0.29 | 4.54 ± 0.21 |  |
| Day 90 | 4.49 ± 0.26 | 4.55 ± 0.27 |  |
| Total Globulin (g/dL) |  |  |  |
| Day 0 | 2.38 ± 0.34 | 2.43 ± 0.32 | 0.701 |
| Day 7 | 2.40 ± 0.35 | 2.36 ± 0.30 |  |
| Day 30 | 2.44 ± 0.39 | 2.36 ± 0.30 |  |
| Day 90 | 2.49 ± 0.38 | 2.41 ± 0.33 |  |
| Albumin:Globulin |  |  |  |
| Day 0 | 1.97 ± 0.32 | 1.94 ± 0.30 | 0.611 |
| Day 7 | 1.92 ± 0.31 | 1.97 ± 0.33 |  |
| Day 30 | 1.87 ± 0.31 | 1.95 ± 0.28 |  |
| Day 90 | 1.86 ± 0.34 | 1.93 ± 0.32 |  |
| Total Bilirubin (mg/dL) |  |  |  |
| Day 0 | 0.66 ± 0.41 | 0.65 ± 0.40 | 0.517 |
| Day 7 | 0.64 ± 0.52 | 0.57 ± 0.31 |  |
| Day 30 | 0.62 ± 0.49 | 0.58 ± 0.31 |  |
| Day 90 | 0.59 ± 0.38 | 0.60 ± 0.32 |  |
| Alkaline Phosphatase (IU/L) | |  |  |
| Day 0 | 68.7 ± 16.1 | 70.0 ± 16.2 | 0.803 |
| Day 7 | 67.2 ± 15.9 | 70.2 ± 15.4 |  |
| Day 30 | 72.0 ± 33.7 | 69.7 ± 15.3 |  |
| Day 90 | 66.9 ± 15.6 | 69.6 ± 15.7 |  |
| Aspartate Aminotransferase (IU/L) | |  |  |
| Day 0 | 25.4 ± 12.5 | 26.1 ± 20.3 | 0.941 |
| Day 7 | 23.5 ± 8.2 | 22.7 ± 8.2 |  |
| Day 30 | 26.2 ± 17.9 | 24.2 ± 11.0 |  |
| Day 90 | 23.9 ± 10.2 | 25.4 ± 11.0 |  |
| Alanine Aminotransferase (IU/L) | |  |  |
| Day 0 | 24.3 ± 15.8 | 25.4 ± 20.2 | 0.819 |
| Day 7 | 22.5 ± 13.6 | 23.4 ± 19.7 |  |
| Day 30 | 27.9 ± 28.5 | 25.5 ± 17.5 |  |
| Day 90 | 24.2 ± 13.8 | 25.2 ± 16.1 |  |
| Abbreviations: SD: standard deviation; PSE: proprietary spearmint extract; PLA: placebo. Treatment P values are shown. +SD is unavailable due to a single measurement value. | | | |

Supplemental Table S3. Blood Lipids.

| **Qu Outcome** | **PLA**  **Mean ±** **SD** | **PSE**  **Mean ±** **SD** | **P-value** |
| --- | --- | --- | --- |
| Total Cholesterol (mg/dL) |  |  |  |
| Day 0 | 169.7 ± 32.1 | 166.4 ± 28.9 | 0.976 |
| Day 7 | 164.0 ± 30.4 | 164.1 ± 35.2 |  |
| Day 30 | 171.2 ± 34.4 | 168.8 ± 34.9 |  |
| Day 90 | 169.8 ± 35.1 | 171.7 ± 36.8 |  |
| Triglycerides (mg/dL) |  |  |  |
| Day 0 | 82.4 ± 47.1 | 98.5 ± 92.0 | 0.107 |
| Day 7 | 82.5 ± 56.6 | 109.7 ± 126.8 |  |
| Day 30 | 87.6 ± 45.8 | 97.3 ± 80.2 |  |
| Day 90 | 82.6 ± 46.8 | 115.5 ± 146.6 |  |
| High Density Lipoprotein Cholesterol (HDL, mg/dL) | |  |  |
| Day 0 | 56.8 ± 16.0 | 54.7 ± 13.0 | 0.365 |
| Day 7 | 57.5 ± 15.4 | 52.5 ± 13.8 |  |
| Day 30 | 57.6 ± 14.2 | 52.3 ± 14.2 |  |
| Day 90 | 57.7 ± 15.0 | 52.9 ± 14.2 |  |
| Very Low Density Lipoprotein Cholesterol (VLDL, mg/dL) | | |  |
| Day 0 | 16.4 ± 9.4 | 17.0 ± 9.1 | 0.567 |
| Day 7 | 16.5 ± 11.3 | 16.6 ± 7.4 |  |
| Day 30 | 17.5 ± 9.2 | 17.8 ± 10.4 |  |
| Day 90 | 16.5 ± 9.4 | 17.8 ± 8.6 |  |
| Low Density Lipoprotein (LDL, mg/dL) | |  |  |
| Day 0 | 96.4 ± 29.2 | 93.5 ± 23.1 | 0.950 |
| Day 7 | 89.8 ± 25.8 | 91.7 ± 27.0 |  |
| Day 30 | 96.1 ± 30.4 | 97.0 ± 27.9 |  |
| Day 90 | 95.6 ± 29.3 | 95.7 ± 26.7 |  |
| LDL:HDL |  |  |  |
| Day 0 | 1.98 ± 1.6 | 1.79 ± 0.69 | 0.563 |
| Day 7 | 1.70 ± 0.77 | 1.84 ± 0.79 |  |
| Day 30 | 1.79 ± 0.79 | 2.02 ± 1.1 |  |
| Day 90 | 1.80 ± 0.80 | 1.88 ± 0.74 |  |
| Abbreviations: SD: standard deviation; PSE: proprietary spearmint extract; PLA: placebo. Treatment P values are shown. | | | |

Supplemental Table S4. Vitals.

| **Qu Outcome** | **PLA**  **Mean ±** **SD** | **PSE**  **Mean ±** **SD** | **P-value** |
| --- | --- | --- | --- |
| Weight (kg) |  |  |  |
| Day 0 | 78.277 ± 15.68 | 79.130 ± 13.27 | 0.768 |
| Day 7 | 77.831 ± 16.65 | 78.441 ± 13.74 |  |
| Day 30 | 78.484 ± 17.12 | 79.449 ± 13.73 |  |
| Day 90 | 77.708 ± 16.45 | 79.353 ± 13.33 |  |
| Body Mass Index (kg/m^2^) |  |  |  |
| Day 0 | 25.831 ± 3.32 | 25.925 ± 3.18 | 0.885 |
| Day 7 | 25.630 ± 3.54 | 25.844 ± 3.34 |  |
| Day 30 | 25.823 ± 3.64 | 26.028 ± 3.39 |  |
| Day 90 | 25.720 ± 3.59 | 25.987 ± 3.61 |  |
| Systolic Blood Pressure |  |  |  |
| Day 0 | 114.72 ± 13.1 | 114.02 ± 11.9 | 0.438 |
| Day 7 | 112.53 ± 11.9 | 112.38 ± 12.4 |  |
| Day 30 | 110.79 ± 12.7 | 114.40 ± 11.0 |  |
| Day 90 | 113.23 ± 12.4 | 116.14 ± 11.2 |  |
| Diastolic Blood Pressure |  |  |  |
| Day 0 | 72.65 ± 8.8 | 72.84 ± 8.3 | 0.197 |
| Day 7 | 70.70 ± 9.5 | 72.90 ± 9.9 |  |
| Day 30 | 70.54 ± 9.7 | 74.45 ± 8.4 |  |
| Day 90 | 73.40 ± 9.0 | 74.50 ± 8.6 |  |
| Heart Rate |  |  |  |
| Day 0 | 63.33 ± 10.2 | 65.64 ± 10.8 | 0.092 |
| Day 7 | 60.83 ± 8.7 | 64.27 ± 10.5 |  |
| Day 30 | 61.92 ± 8.5 | 64.54 ± 10.1 |  |
| Day 90 | 62.36 ± 8.8 | 63.87 ± 9.7 |  |
| Abbreviations: SD: standard deviation; PSE: proprietary spearmint extract; PLA: placebo. Treatment P values are shown. | | | |
